# Can a Giant Cell Arteritis (GCA) Risk Stratification Score Be Helpful in Clinical Practice?

**DOI:** 10.7759/cureus.32310

**Published:** 2022-12-08

**Authors:** Muhamad Jasim, Priyan Magan, Ferin Patel, Tochukwu Adizie, Dhanuja Senn

**Affiliations:** 1 Rheumatology, New Cross Hospital, Wolverhampton, GBR; 2 General Practice, Russells Hall Hospital, Birmingham, GBR; 3 Plastic Surgery, New Cross Hospital, Wolverhampton, GBR

**Keywords:** ultrasound temporal artery, temporal artery biopsy, probability score, giant cell arteritis, gca

## Abstract

Introduction: Giant cell arteritis (GCA) is the most common type of large vessel vasculitis. The diagnosis of GCA is often challenging and there is a difficult balance of over- and underinvestigation. There have been several proposed scoring systems to help clinicians risk stratify patients who may present with suspected GCA.

Methods: A retrospective cohort study was performed using electronic medical records of patients referred for a temporal artery biopsy (TAB) and temporal artery ultrasound scan (USS) for suspected GCA. All TABs performed at the Royal Wolverhampton NHS Trust between June 2014 and June 2018 and all USS procedures performed between January 2015 and January 2019 were analysed. Patients who undergo a USS for suspected GCA at our centre routinely have scanned bilateral temporal and axillary arteries. Patients were excluded if they already had a previous diagnosis of GCA (and the clinical question was suspected flare), or if there was insufficient information available.

Results: The total number of patients who underwent a confirmatory diagnostic test (either TAB or USS) for suspected GCA was 187. Thirteen of these patients met the exclusion criteria, the remaining 174 patients were included for analysis. A total of 126 of 174 patients underwent a TAB and 63 of 174 had a USS performed; 15 of 174 who had both these were included in the USS cohort because for all these patients, the ultrasound was the first diagnostic test performed. Our results appear to closely mirror the original multi-centre results with regard to the prediction of biopsy-positive GCA, with the centiles closely following those in the inception cohort. Also, 0% of the ‘low’ risk probability biopsy cohort were misclassified; none had a positive biopsy. However, 8% of the low-risk-probability ultrasound cohort were misclassified, as two had a positive ultrasound.

Conclusion: Our study highlights that a probability score for GCA derived from a large multi-centre cohort of patients who were biopsy positive predicts ultrasound positivity with similar accuracy. Our work reveals that scoring systems are not infallible but can be helpful in guiding clinical decision making.

## Introduction

Giant cell arteritis (GCA) is the most common type of large vessel vasculitis [1]. Typically, it presents in patients over the age of 50 with a combination of temporal headaches, scalp tenderness, jaw claudication, raised inflammatory markers and visual disturbance [2]. The diagnosis of GCA is often challenging and there is a difficult balance of over- and underinvestigation. On the one hand, there are numerous mimics of GCA, while on the other hand, there is a significant risk of visual loss. Overinvestigation and treatment may lead to unnecessary invasive procedures (e.g. temporal artery biopsy, or TAB) and unnecessary steroid burden and underinvestigation may risk missed cases with possible permanent consequences. There have been several proposed scoring systems to help clinicians risk stratify patients who may present with suspected GCA [3]. One such scoring system, published in 2017, showed clinical utility in a large international multi-centre study. Following analysis by logistic regression on data from 530 biopsies, Ing et al. developed a parsimonious prediction model comprising five candidate criteria: age, jaw claudication, ischemia-related loss of visual acuity, platelet count, and log C-reactive protein (CRP) [4].

Increasingly, ultrasound Doppler imaging is being accepted as the satisfactory confirmation of the diagnosis of GCA, with the presence of the halo sign characteristic for GCA [5]. The aim of our study was to determine whether this GCA prediction model accurately predicts positive TABs in a large, real world UK cohort. In addition, we assessed whether this model accurately predicts a positive temporal artery ultrasound.

## Materials and methods

A retrospective study was performed using electronic medical records of patients referred for a temporal artery biopsy and temporal artery ultrasound scan (USS) for suspected giant cell arteritis. These two groups were analysed separately. All temporal artery biopsies performed at the Royal Wolverhampton NHS Trust between June 2014 and June 2018 and all temporal artery ultrasound scans performed between January 2015 and January 2019 were analysed. The lists of temporal artery biopsies and ultrasounds were obtained from the histology lab and radiology department. Patients who undergo a USS for suspected GCA at our centre routinely have bilateral temporal and axillary arteries scanned. Patients were excluded if they already had a previous diagnosis of GCA or if there was insufficient information available.

## Results

The total number of patients who underwent a confirmatory diagnostic test (either temporal artery ultrasound or biopsy) for suspected GCA was 187. Four biopsies and nine ultrasounds were excluded due to insufficient information or because they were done to rule out a flare of GCA rather than for diagnostic purposes. Therefore, 174 patients were included for analysis. Of them, there were 111 patients who only underwent a biopsy; 63 patients underwent a USS, of whom 17 patients had both a USS and TAB, and these were included in the USS cohort because for all these patients, ultrasound was the first diagnostic test performed.

A total of 75 (68%) biopsies were requested by the ophthalmology department, 26 (23%) were requested by the rheumatology department, and the remaining 9% of TABs were requested by other clinicians, for example, neurologists or general physicians. All other temporal artery USS procedures were requested by the rheumatology department.

Of the 63 ultrasound patients, 24 had positive ultrasound scans and all except one patient were treated as GCA cases. Six of these patients also had TABs, two of which were negative and four were positive. A total of 39 had negative ultrasounds; 11 of this cohort went on to have biopsies. Of these biopsies, two were positive and these patients were treated as having GCA. One of these patients had an intermediate probability score (0.2) and the other had a high score (0.65). Both of these patients had been on steroids for more than a week prior to the USS procedure.

Among the 111 TAB patients, 32 had a positive TAB. Two had biopsies noted to have features of ‘healed arteritis’, which we classed as a positive result. Also, 76 TABs were negative and four TABs were inconclusive (also classified as negative). The mean length of biopsy samples in our study was 11 mm (range 4-34 mm). The mean age of our TAB cohort was 71.7 years, while in the USS cohort, it was 71.1 years. A total of 63% cases of our TAB cohort were female compared to 79% of the USS cohort.

Overall, 85 of the 174 patients (49%) were treated for GCA. A total of 56 of 85 (66%) patients had a positive USS and/or a positive TAB. The remaining 29 of 85 (34%) were treated based on a clinical diagnosis: 21 of 29 had a negative TAB only, 5 of 29 had a negative TAB and USS, and 3 of 29 had a negative USS and refused/did not have a TAB. Three patients were found to have a positive TAB or USS despite being ‘seronegative’, i.e., having a normal platelet count (<450 per microliter), erythrocyte sedimentation rate (ESR) <50 mm/hour, and adjusted CRP ≤5 mg/l. Of these three patients, one patient had biopsy-proven GCA (1 of 31, 3.2%). The 77-year-old man had visual loss and headache with no other symptoms and a GCA risk probability score of 0.08. The second patient had ultrasound-positive GCA. This was a 63-year-old lady who presented with bilateral temporal region headache, reduced vision, and scalp tenderness, with a probability score of 0.06. Third was a 66-year-old man with temporal headache but no other symptoms. He initially had a positive USS but went on to have a negative TAB, and was not treated as GCA. His risk probability score was 0.045. Results are summarised in Tables 1-3.

**Table 1:** Investigation outcome summary TAB, temporal artery biopsy; USS, ultrasound scan; GCA, giant cell arteritis ^a^Of these, 17 patients had both USS and TAB.

TAB, n = 111				USS, n = 63^a^			
Positive TAB, n = 31 (28%)		Negative TAB, n = 80 (72%)		Positive USS, n = 24 (38%)		Negative USS, n = 39 (62%)	
30 positive	1 healed GCA, classified as positive	76 negative	4 inconclusive, classified as negative	19 positive	5 with partially/fully treated GCA	38 negative	1 indeterminate (didn’t undergo TAB)
				6 had TAB: 4 of 6, +ve TAB; 2 of 6, –ve TAB; 5 of 6 patients were treated as GCA	None of these had TAB. All were treated as GCA	11 of these patients went on to have a TAB: 9 of 11, –ve TAB; 2 of 11, +ve TAB	

**Table 2:** Data analysis TAB, temporal artery biopsy; USS, ultrasound scan

Total no. of patients = 174	TAB cohort	USS cohort
Number of patients	111	63
Mean age	71.7	71.1
Female:male ratio (%)	70:41 (63% F)	49:13 (79% F)
Overall score, mean	15.7	15.2
Overall score, range	23.7–9.5	22.45–9.3
Risk probability median	0.27	0.21
Risk probability mean	0.33	0.31
Mean positive TAB/USS probability score	0.51	0.41
Highest probability score for positive TAB/USS	0.95	0.87
Lowest probability score for positive TAB/USS	0.08	0.045
	0.14	0.06
	0.15	0.13

**Table 3:** Distribution of patient cohorts between the probability score cutoff points TAB, temporal artery biopsy; USS, ultrasound scan; GCA, giant cell arteritis; RWT, Royal Wolverhampton Trust This is a comparison with Ing et al.'s multi-centre study results [4].

Distribution				
GCA probability score	Low <0.07	Intermediate 0.07–0.42	High 0.43–0.88	Very high ≥0.89
Multi-centre study, TAB +ve	<5^th^ centile	5^th^–50^th^ centile	50^th^–96^th^ centile	>96^th^ centile
RWT TAB +ve, n = 32	0	12	17	2
		0–39^th^ centile	39^th^–94^th^ centile	>94^th^ centile
RWT USS +ve, n = 24	2	12	10	0
	<8^th^ centile	8^th^–58^th^ centile	>58^th^ centile	
GCA probability score	Low <0.07	Intermediate 0.07–0.42	High 0.43–0.88	Very high ≥0.89
Multi-centre study, TAB -ve	<30^th^ centile	30^th^–91^st^ centile	92^nd^–99^th^ centile	
RWT TAB -ve, n = 79	11	53	16	0
	<14^th^ centile	14^th^–80^th^ centile	>80^th^ centile	
RWT USS -ve, n = 39	8	25	5	1
	<21^st^ centile	21^st^–85^th^ centile	85^th^–98^th^ centile	>98^th^

**Figure 1:**
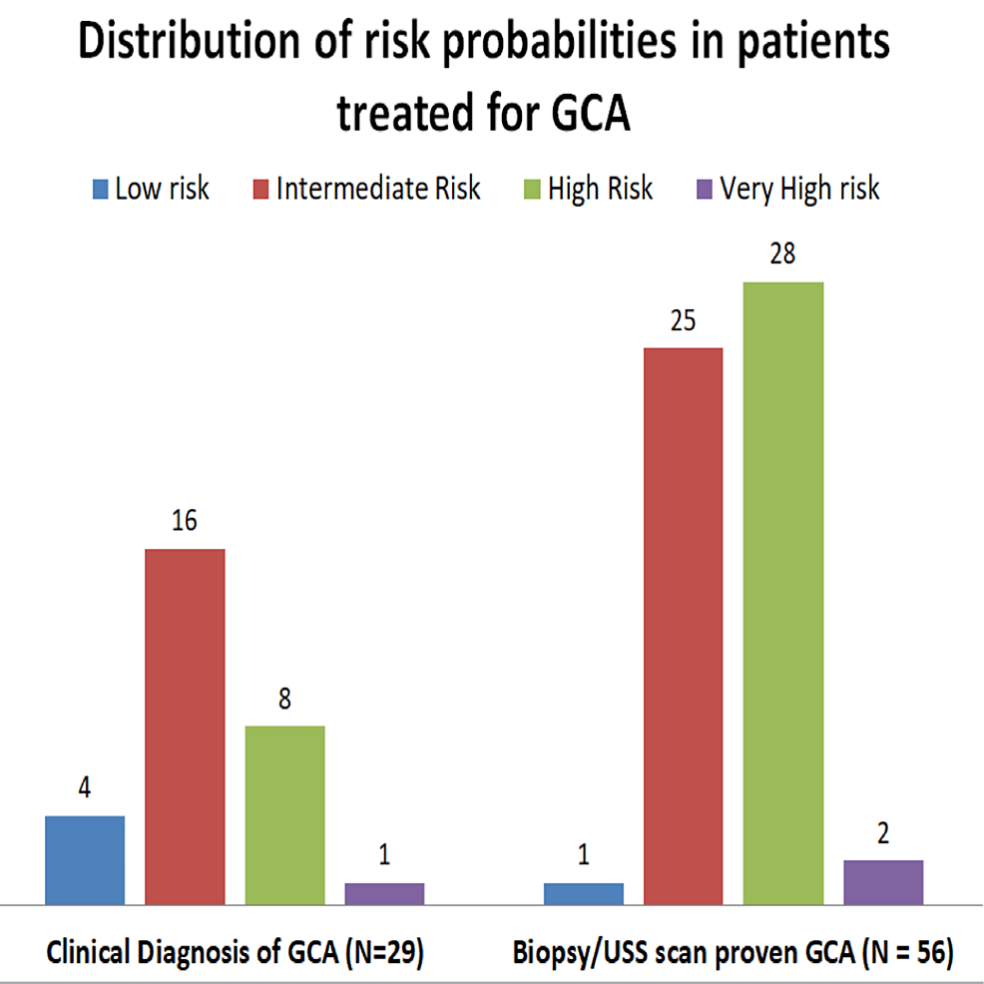
GCA risk probability distribution in patients treated as GCA GCA, giant cell arteritis

Figure 1 illustrates the distribution of the risk probability scores in the 85 patients treated for GCA. 

## Discussion

It should be highlighted that we grouped several of the probability score cutoffs. Hence, in total we had four probability groups (low, intermediate, high and very high risk) in comparison to the seven groups of the multi-centre study. This was for better real-world clinical utility and usability.

GCA is the commonest systemic vasculitis and has a varied presentation. Furthermore, existing classification criteria are inadequate for diagnosis. The benefits of a validated probability score feasible in routine clinical practice are threefold. Although TAB is usually a benign test, it is invasive and time-consuming. Ideally risk calculators should predict the risk of GCA prior to TAB to guide decision making. With the increasing use of ultrasound, a reliable risk calculator in combination with a non-invasive test would ideally reduce the requirement of temporal artery biopsies substantially. Secondly, while rapid initiation of treatment is crucial to prevent visual loss, such a score would also be beneficial because it would actually serve to increase clinician confidence in stopping inappropriate glucocorticoids in those who do not have GCA, given that 85% of patients experience glucocorticoid-related side effects [6]. Finally, though rare, mimics of GCA on ultrasound have been identified, and a low probability score in combination with unexpectedly positive ultrasound would guide clinicians in seeking histological confirmation of the diagnosis with a biopsy [7]. Our results appear to closely mirror the original multi-centre study results with regard to the prediction of biopsy-positive GCA, with the centiles closely following those in the inception cohort. The 2018 European Alliance of Associations for Rheumatology (EULAR) guidance stipulates that the temporal artery ultrasound is the first-line investigation in centres with appropriate expertise [8]. A further reason for including ultrasound as well as biopsy-proven GCA patients relates to commissioning guidance regarding the use of biologics for GCA in the UK. Evidence of a positive confirmatory investigation (imaging or histology) or ischemic complication of GCA is required for patients to be eligible for tocilizumab in the National Health Service (or NHS) [9]. Moreover, ultrasound is more readily incorporated into a system of fast track clinics for GCA, which is fast becoming the gold standard method of assessment and treatment of patients referred with suspected GCA.

There have been several attempts to create probability scores in GCA. The strength of this score is the large sample size of the cohort from which it was derived, the brevity of the score (only five variables), and the fact that an online calculator has been created. It was derived in an ophthalmology setting; in this study, we have demonstrated that it is valid in a mixed rheumatology and ophthalmology cohort of suspected GCA patients, indicating that it has generalisability. Limitations of our study include its retrospective nature with missing data, the constraint to biopsy- or ultrasound-positive GCA, and misclassification rate. It is well recognised that a proportion of patients in routine clinical practice will have ‘biopsy-negative GCA’ due to skip lesions, prolonged steroid treatment, or inadequate sample length. The same concerns apply to ultrasound, and in fact, ultrasound is far more sensitive to steroid therapy, with ultrasound vessel wall abnormalities frequently disappearing after 48-72 hours of high-dose glucocorticoids [10]. Due to incomplete documentation, we were unable derive the mean number of days on steroids before biopsy and ultrasound. The challenge is that there are no independent validating criteria to determine whether giant cell arteritis is present when a temporal artery biopsy is negative, and in this setting, the diagnosis of GCA rests on clinical presentation, exclusion of mimics, and response to steroids.

Our work reveals that scoring systems are not infallible but can be helpful in guiding clinical decision making (Table 4). It is worth highlighting that the presence of visual disturbance and temporal headache even with normal inflammatory markers and a low risk probability score should increase the suspicion of GCA. In such cases, an urgent review from the ophthalmology department is important.

**Table 4:** Applying the probability score to our local GCA guidelines TAB, temporal artery biopsy; USS, ultrasound scan; GCA, giant cell arteritis; PPI, proton pump inhibitor

	GCA probability score			
	Low <0.07	Intermediate 0.07-0.42	High 0.43-0.88	Very high ≥0.89
Medication to commence	Consider need for steroids, bone protection and PPI	Start steroids, bone protection and PPI	Start steroids, bone protection and PPI		
Decision for USS	Arrange urgent temporal artery USS (call the USS department)	Arrange urgent temporal artery USS (call the USS department)	Arrange urgent temporal artery USS (call the USS department)		
Decision on TAB	GCA diagnosis highly unlikely if USS negative; look for different diagnosis	If USS negative, consider the need and discuss the option of TAB	If USS negative, arrange for TAB		
Final treatment decision	If USS positive but atypical presentation, discuss TAB	If positive, treat as GCA	If positive, treat as GCA		

This article was previously published as an abstract in the British Medial Journal, Annals of Rheumatic Diseases [11].

## Conclusions

Our study highlights that a probability score for GCA derived from a large multi-centre cohort of ophthalmology patients who were biopsy positive predicts ultrasound positivity with similar accuracy to biopsy positivity. We have also demonstrated that this probability score performs well in a mixed cohort of ophthalmology and rheumatology patients. Our work reveals that scoring systems are not infallible but can be helpful in guiding clinical decision making.
